# Generation of Hydrogen Peroxide in Cancer Cells: Advancing Therapeutic Approaches for Cancer Treatment

**DOI:** 10.3390/cancers16122171

**Published:** 2024-06-07

**Authors:** Taufeeque Ali, Daniel Li, Thilini Nimasha Fernando Ponnamperumage, Alexis Kimberly Peterson, Jatin Pandey, Kulsum Fatima, John Brzezinski, Julia Anna Rose Jakusz, Hanlun Gao, Gilbert Edward Koelsch, Dhivyashree Senthil Murugan, Xiaohua Peng

**Affiliations:** Department of Chemistry and Biochemistry and the Milwaukee Institute for Drug Discovery, University of Wisconsin-Milwaukee, 3210 N. Cramer Street, Milwaukee, WI 53211, USA; alit@uwm.edu (T.A.); thevengefuldolphin@gmail.com (D.L.); pnf@uwm.edu (T.N.F.P.); pete2358@uwm.edu (A.K.P.); jpandey@uwm.edu (J.P.); kulsumfatima724@gmail.com (K.F.); brzezi36@uwm.edu (J.B.); jajakusz@uwm.edu (J.A.R.J.); hanlun@uwm.edu (H.G.); gkoelsch@uwm.edu (G.E.K.); senthil4@uwm.edu (D.S.M.)

**Keywords:** Prooxidants, hydrogen peroxide generation, reactive oxygen species, phenol and polyphenols, quinones, metal peroxides, ascorbic acid, anticancer drugs, tumor-selective therapeutic strategy

## Abstract

**Simple Summary:**

Cancer cells grow and divide so rapidly that they are literally engaged in a metabolic marathon—leading to the formation of high levels of reactive oxygen species (ROS). The altered redox balance in cancer cells offers therapeutic opportunities for targeting cancer. Some ROS-targeting strategies attempt to enhance ROS production to inflict lethal cell damage or trigger apoptosis, while other anticancer agents inhibit enzymes that are essential to maintain the redox potential of the cell. One type of ROS that shows potential to be utilized as a treatment mechanism is hydrogen peroxide (H_2_O_2_), which can be generated by numerous mechanisms, including metal-based prodrugs, photodynamic therapy, enzymes, nanoparticles, chemical modulators, and various combinations thereof. This review focuses on various chemical agents that induce H_2_O_2_-mediated cancer cell death, including their mechanism of function, preclinical and clinical studies, recent advancements, and potential applications as a new and exciting avenue for targeted and efficacious cancer treatment.

**Abstract:**

Cancer cells show altered antioxidant defense systems, dysregulated redox signaling, and increased generation of reactive oxygen species (ROS). Targeting cancer cells through ROS-mediated mechanisms has emerged as a significant therapeutic strategy due to its implications in cancer progression, survival, and resistance. Extensive research has focused on selective generation of H_2_O_2_ in cancer cells for selective cancer cell killing by employing various strategies such as metal-based prodrugs, photodynamic therapy, enzyme-based systems, nano-particle mediated approaches, chemical modulators, and combination therapies. Many of these H_2_O_2_-amplifying approaches have demonstrated promising anticancer effects and selectivity in preclinical investigations. They selectively induce cytotoxicity in cancer cells while sparing normal cells, sensitize resistant cells, and modulate the tumor microenvironment. However, challenges remain in achieving selectivity, addressing tumor heterogeneity, ensuring efficient delivery, and managing safety and toxicity. To address those issues, H_2_O_2_-generating agents have been combined with other treatments leading to optimized combination therapies. This review focuses on various chemical agents/approaches that kill cancer cells via H_2_O_2_-mediated mechanisms. Different categories of compounds that selectively generate H_2_O_2_ in cancer cells are summarized, their underlying mechanisms and function are elucidated, preclinical and clinical studies as well as recent advancements are discussed, and their prospects as targeted therapeutic agents and their therapeutic utility in combination with other treatments are explored. By understanding the potential of these compounds, researchers can pave the way for the development of effective and personalized cancer treatments.

## 1. Introduction

Cancer is a complex and multifaceted disease that poses immense challenges for researchers around the globe to develop targeted therapies. Serious side-effects are common with current cancer therapies due to their lack of cellular specificity. Extensive efforts have been made to develop more selective therapeutics to specifically kill cancer cells, and a major focus is to exploit the inherent vulnerabilities among cancer cells in order to improve the selectivity of cancer treatment. One such approach involves manipulation of reactive oxygen species (ROS), due to the fact that cancer cells are known to exhibit increased intrinsic oxidative stress compared to normal cells [1,2,3,4,5]. This intrinsic feature of cancer paves the way for the development of tumor-selective therapeutic strategies [3,6,7,8,9]. This review specifically focuses on strategies to selectively modulate the most stable ROS, hydrogen peroxide (H_2_O_2_), in cancer cells to achieve therapeutic effects.

**Figure 1:**
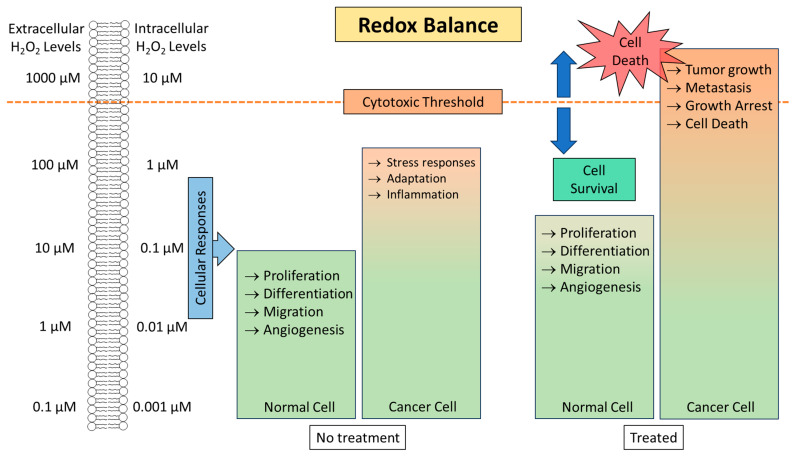
The redox imbalance of cancer cells and its therapeutic potential. The elevated levels of H_2_O_2_ in cancer cells trigger a redox adaptation response which further increase their antioxidant capacity to maintain H_2_O_2_ levels near the cytotoxic threshold and allow selectively targeting cancer cells by prooxidants that push H_2_O_2_ levels beyond the threshold and inducing cell death.

Mildly increased levels of H_2_O_2_ are known to contribute to tumor growth by promoting the transformation, proliferation, and survival of cancerous cells, as well as angiogenesis and metastasis. High levels of H_2_O_2_ can cause damage to various biomolecules, such as DNA, proteins, and lipids, and thus are detrimental to cell survival and growth [5,10]. Although cancer cells produce large quantities of H_2_O_2_, their natural H_2_O_2_ levels are not sufficient to achieve noticeable therapeutic results. Amplification of H_2_O_2_ production, specifically within cancer cells, has emerged as a valuable approach for cancer treatment [7,10]. In healthy somatic cells, ROS generation and elimination are in a delicate equilibrium, and thus a low level of basal ROS is achieved, ensuring redox homeostasis. This allows them to quickly adapt to any changes in oxidative stress. However, due to heightened metabolic irregularities and increased oncogenic signaling, cancer cells have high levels of H_2_O_2_ to begin with, which can trigger redox adaptation and lead to upregulation of antioxidant concentrations, such as glutathione (GSH) and thioredoxin. This further increases the redox capacity of the cancer cells, thus allowing them to maintain high levels of H_2_O_2_ that are very close to the cytotoxic threshold (Figure 1). Consequently, cancer cells are susceptible to prooxidants that generate H_2_O_2_ beyond the cytotoxic threshold, which leads to cell death [11]. This phenomenon provides a biochemical foundation for developing therapeutics that selectively target cancer cells through H_2_O_2_-mediated mechanisms. Various strategies have been developed to boost H_2_O_2_ levels specifically within cancer cells to achieve selective therapeutic effects, such as treatment with glucose oxidase, lactate oxidase, peroxides, H_2_O_2_-releasing chemical agents, inhibitors of antioxidative enzymes, such as superoxide dismutase (SOD), glutathione peroxidase (GPx), and catalase that eliminate H_2_O_2_ in cells to maintain homeostasis. This review focuses on various chemical agents that kill cancer cells via selective H_2_O_2_ generation. This paper, which is not meant to be a thorough review, will discuss different categories of H_2_O_2_-producing agents and the mechanism underlying H_2_O_2_ production through a few selected examples.

## 2. Hydrogen Peroxide Generation and Its Reactivity with Biomolecules

**Figure 2:**
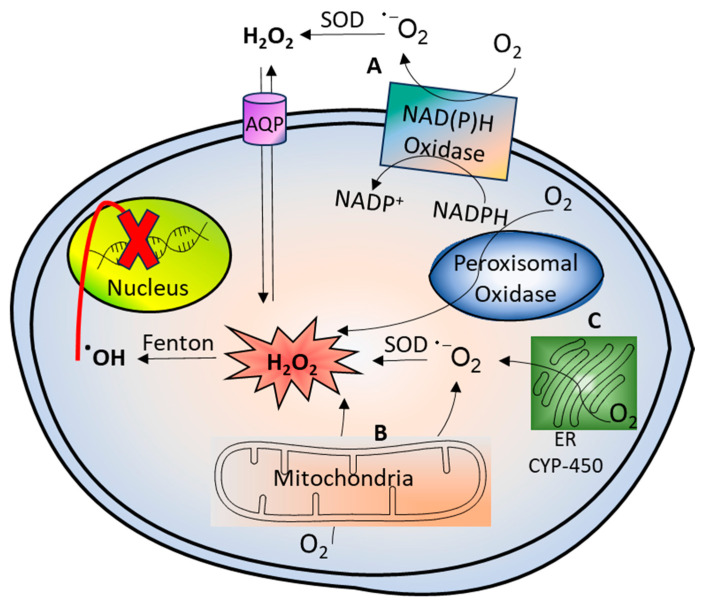
Three major pathways for cellular H_2_O_2_ production: (A) NADPH catalyzed conversion of O_2_ to superoxide radical anion (O_2_^•−^), followed by transformation of O_2_^•−^ to H_2_O_2_ via SOD. (B) Involvement of the mitochondrial respiratory chain, where cytochrome complex generates O_2_^•−^. (C) H_2_O_2_ production mediated by the oxidases and endoplasmic reticulum.

H_2_O_2_ is naturally formed in living organisms and plays an important role as a redox metabolite that is involved in redox signaling, sensing, and regulation [7,12]. It functions as a messenger molecule that permeates through cells and tissues, triggering rapid cellular effects such as alteration in cell shape, cellular proliferation, and recruitment of immune responses. There are three major pathways for H_2_O_2_ formation in normal mammalian cells, all of which involve the reduction of O_2_ into superoxide anion (O_2_^•−^) (Figure 2) [13]. The first pathway involves the nicotinamide adenine dinucleotide phosphate (NADPH) oxidase that catalyzes the conversion of oxygen (O_2_) into O_2_^•−^, which then dismutates to H_2_O_2_ by SOD. The second pathway pertains to the mitochondrial respiratory chain, particularly the cytochrome complex, in the production of superoxide radicals. The third mechanism involves the action of oxidases in specific cell types or subcellular organelles, such as xanthine oxidase, glucose oxidase, monoamine oxidase, or D-amino acid oxidase. Another cellular compartment that contributes to H_2_O_2_ production is the endoplasmic reticulum [14].

**Figure 3:**
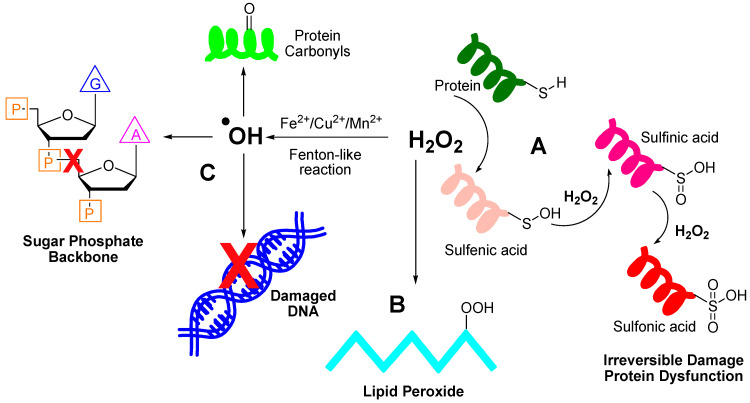
Damaging effects of H_2_O_2_ to biomolecules via several biochemical reactions: (**A**) direct oxidation of the thiol groups by H_2_O_2_; (**B**) direct lipid oxidation by H_2_O_2_; (**C**) conversion of H_2_O_2_ to highly reactive •OH via the Fenton-like reaction, which induces sugar phosphate backbone cleavage, nucleobase, or protein modification.

In a biological context, H_2_O_2_ causes damage to biomolecules via several biochemical reactions. It can oxidize thiol groups such as cysteine-containing proteins, forming sulfenic acids, which can further oxidize to form sulfinic and sulfonic acids, causing permanent protein inactivation (Figure 3) [15]. However, the chemical reactivity of H_2_O_2_ alone towards biomolecules remains low. In the presence of transition metal ions such as Fe^2+^, Cu^+^, Mn^2+^, etc., H_2_O_2_ is converted to highly reactive hydroxyl radical (^•^OH) via the Fenton-like reaction, which causes damage to both DNA and proteins. Hydroxyl radicals can cleave the sugar phosphate backbone and modify the nucleobases, such as guanine and thymine. Proteins are also very vulnerable to ^•^OH attack, especially those with thiol groups like methionine. Hydroxyl radicals also interact with several other amino acids such as lysine, proline, arginine, and histidine, forming protein carbonyls and 2-oxo-histidines, compromising protein functionality. The collective impact of this oxidative damage results in heightened oxidative stress on several components of the cells, which is detrimental to the cell and often leads to cell death [16].

## 3. Concentration-Dependent Selection between Apoptosis and Necrosis Induced by H_2_O_2_

**Figure 4:**
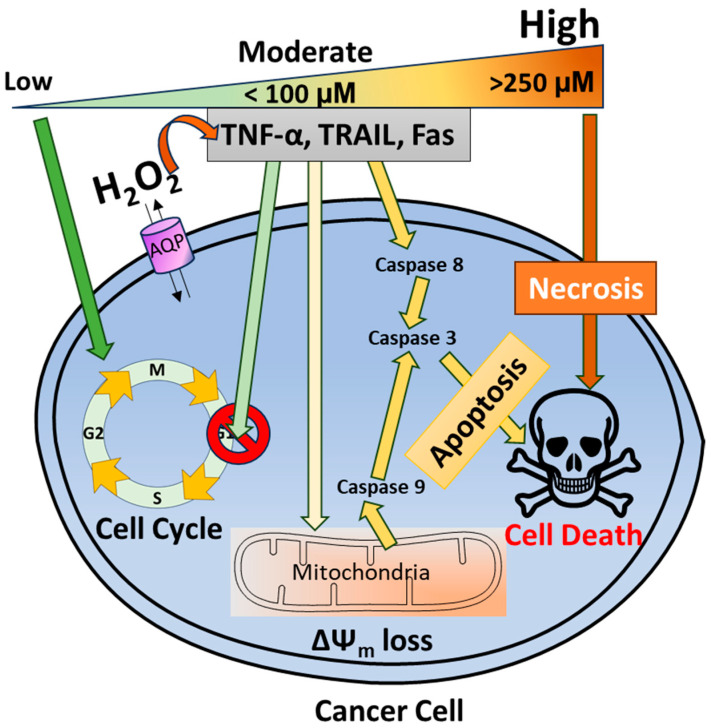
Concentration-dependent cell death pathways induced by H_2_O_2_ (apoptosis induced by low or moderate H_2_O_2_ and necrosis induced by high concentrations of H_2_O_2_).

Two distinct pathways, apoptosis and necrosis, have been reported for H_2_O_2_-induced cell death (Figure 4) [17,18,19,20,21,22,23]. H_2_O_2_ concentrations and the exposure duration of cells to H_2_O_2_ can greatly affect the mechanism of cell death. Moderate concentrations of H_2_O_2_ typically induce apoptosis, while higher concentrations induce necrosis [18] (Figure 4). Apoptosis can be induced by acute exposure to moderate H_2_O_2_ levels through intrinsic mitochondrial pathways or extrinsic receptor-mediated pathways [24]. In the case of mitochondrial pathways, H_2_O_2_ prompts the relocation of cytochrome c from the mitochondrion into the cytosol, where it engages with Apaf-1 and caspase-9 to form the apoptosome complex. This complex activates downstream effector caspases (caspase-3, -6, and -7), culminating in cell death via apoptosis [25]. Conversely, the extrinsic pathway entails the binding of specific ligands (e.g., TNF-α, TRAIL, Fas) to death receptors on the cell surface, initiating the activation of caspase-8 [26]. Caspase-8 cleaves BID, instigating mitochondrial membrane depolarization and cytochrome c release, ultimately leading to the activation of caspase-3 and apoptosis. Specific markers such as caspase-9 can indicate the initiation of apoptosis [27], while receptor-interacting protein (RIP) is entwined in necroptotic signaling pathways [28]. Elevated concentrations of H_2_O_2_ can precipitate necrotic cell death characterized by cellular swelling, rupturing of the plasma membrane, and conserved nuclear structure [20,29]. Comprehending the diverse mechanisms by which H_2_O_2_ influences cell death is paramount for both fundamental scientific exploration and clinical applications, furnishing invaluable insights into disease etiology and guiding the development of therapeutic interventions.

## 4. Increasing H_2_O_2_ Level as an Anticancer Therapy

While excessive ROS production can stimulate cellular proliferation and genetic instability, it can also trigger apoptosis, suggesting that ROS-mediated mechanisms can be harnessed for cancer treatment [30]. Many cancer therapies induce ROS (i.e., H_2_O_2_) production as a possible mechanism, such as chemotherapy [31], photodynamic therapy [32], radiotherapy [33], and enzyme-based therapies [30,34]. Various chemotherapeutic agents and radiotherapy directly generate ROS, causing apoptosis in cancer cells. Photodynamic therapy uses photons to activate photosensitizers to produce ROS causing cytotoxicity. However, there are still many challenges for these ROS-amplifying therapies, including off-target effects, limited penetration, and safety concerns. Various strategies have been developed to improve cancer specificity and reduce systemic toxicity, such as the use of prooxidants to amplify ROS production selectively in cancer cells [35], selective delivery of ROS-producing agents to cancer cells via nanoparticles [36], gene therapy for encoding enzymes involved in ROS production to obtain targeted ROS production in cancer cells [37], and immunotherapy that harnesses the body’s defenses to recognize and attack cancer cells through ROS production [38]. Collectively, these approaches provide a wide range of applications that include customization, personalization, precision treatment with reduced toxicity, enhanced efficacy, and minimized resistance towards the ongoing battle against cancer.

**Figure 5:**
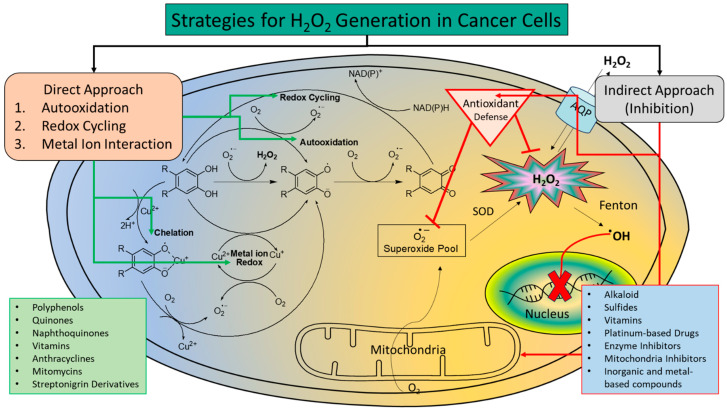
Strategies for H_2_O_2_-induction in cancer cells: Direct approaches include autooxidation, redox cycling, and metal ion interactions whereas indirect approaches include inhibitors of antioxidants defense system such as SOD, GSH, and catalase and inhibitors of cellular organelles responsible for redox balance, such as mitochondria and the endoplasmic reticulum.

The use of prooxidants in cancer therapies has been widespread and gained attention due to various advantages, such as selective cytotoxicity towards cancer cells, reduced off-target effects, and the potential to be used in combination with conventional chemotherapeutic agents to achieve synergistic anticancer effects [7,35]. Their distinct mechanism allows them to overcome drug resistance [3], and the understanding of ROS levels and the antioxidant capacity in individual cancer cells provides an avenue for researchers to tailor treatment strategies and make therapies more personalized and effective [39]. The diverse selection of prooxidants, including natural and synthetic molecules, offer a wide range of options for researchers and clinicians. These compounds hold potential not only because of their direct killing of cancer, but also because of their ability to change H_2_O_2_ levels, thus allowing them to enable synergistic combination therapies with H_2_O_2_-activated prodrugs. Unraveling their potential and understanding their mechanisms of H_2_O_2_ generation will speed up the development of more effective and personalized cancer treatments. Based on the pathways for H_2_O_2_ production, prooxidants fall into two major categories, either the category of directly producing H_2_O_2_ (direct approach) or the category of inhibiting the excessive antioxidative defense system within cancer cells (indirect approach). There are three major mechanisms for direct H_2_O_2_ production induced by prooxidants, including autooxidation, redox cycling, and metal ion interaction (Figure 5). Many prooxidants are directly involved in the electron transport chain for producing H_2_O_2_ in cancer cells, such as phenol and polyphenol analogues, quinone moieties, vitamin C, metal oxides, and many FDA-approved anticancer drugs. They can donate electron/hydrogen atoms for the reduction of molecular O_2_ to O_2_^•−^ and H_2_O_2_ either via autooxidation, redox cycling, or metal ion interaction.

### 4.1. Phenol and Polyphenol Analogues

**Figure 6:**
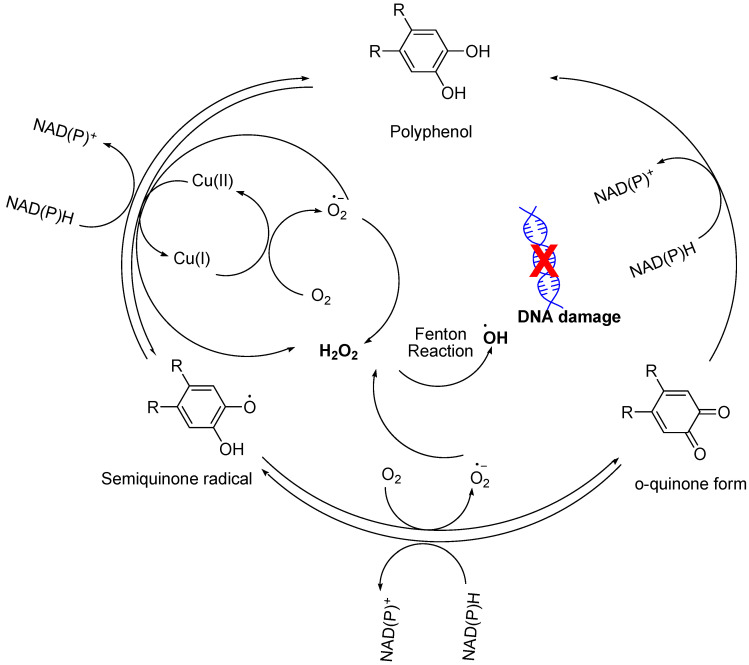
Potential mechanisms for H_2_O_2_ generation induced by compounds containing phenolic groups.

Among various naturally occurring and synthesized compounds, structures with multiple phenolic groups facilitate H_2_O_2_ generation either via repeated steps of autooxidation in the presence of molecular oxygen (O_2_) [40,41], or via redox cycling that involves NAD(P)H (Figure 5 and Figure 6). Autooxidation is often a slow reaction due to the lower redox potential of O_2_/O_2_^•−^ and produces H_2_O_2_ through dismutation. During the autooxidation process, phenols are oxidized to semiquinone, which rapidly transforms into quinone, while O_2_ is reduced to a superoxide radical (O_2_^•−^) that undergoes dismutation to generate H_2_O_2_ at the same time (Figure 6). The formed quinone then undergoes a subsequent reduction in the presence of NAD(P)H enzyme to regenerate polyphenols that spontaneously revert to their quinone form via a semiquinone intermediate. This continuous cycle between oxidation and reduction creates a self-perpetuating cycle known as redox cycling [40]. Compounds that feature polyphenolic (hydroxyl) groups include a wide range of compounds such as flavonoids, hydroxytyrosol, propyl gallate, hydroxycinnamic acids, etc.

#### 4.1.1. Flavonoids

**Figure 7:**
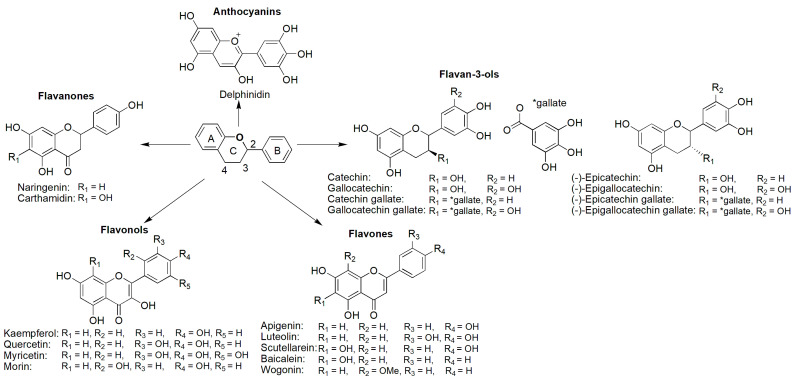
Classification of Prooxidative Flavonoids.

Flavonoids are a diverse set of polyphenolic compounds found in plant-based foods and beverages. They have been extensively explored for their vast range of pharmacological properties such as antibacterial, antimutagenic, antiresorptive, antioxidative, and anticancer effects [42]. A fundamental flavonoid structure includes two benzene rings (A and B) connected by a heterocyclic pyran ring (ring C) (Figure 7). There are five subclasses of flavonoids: flavan-3-ols (such as catechins and gallocatechins), flavones (such as apigenin, luteolin, and baicalein), flavonols (such as kaempferol, quercetin, and myricetin), flavanones (such as naringenin and carthamidin), and anthocyanins (such as delphinidine). These subclasses vary in their structural arrangements of hydroxyl and methoxy groups, and also in their ring conjugations (Figure 7). They have been found to act as prooxidants, and a wide variety of flavonoids (such as catechins [43,44,45,46], baicalein, quercetin, morin, myricetin [47,48,49], and wogonin [50,51,52]) have been reported to produce high level of H_2_O_2_. These flavonoids selectively kill malignant cells via H_2_O_2_ mediated processes and interactions with cellular functions that lead to apoptosis, such as enhanced hydroxyl radical formation via the Fenton reaction, which causes DNA, protein, and cell membrane damage [43,44,45]. The production of H_2_O_2_ has been observed in flavonoid-treated media as well as in cell cultures. Their ability to generate ROS can be influenced by the presence and location of hydroxyl groups [53]. In order to harness their therapeutic benefits while avoiding unintended harmful effects, it is essential to attain a better understanding of their prooxidant activities.

Among various flavonoids, catechins are a popular subclass and have been widely investigated for their prooxidative effects. Catechins belong to the category of flavanols, which have two isomeric forms, a positive (+) form and a negative (−) form (epicatechin). The (+)-catechins have antioxidative properties, whereas the (−)-epicatechins act as pro-oxidants inducing oxidative effects [54]. The presence of phenolic hydroxyl groups in catechins makes them susceptible to repeated redox reactions where these groups donate electrons and form H_2_O_2_ [43]. The process becomes more feasible in ortho-dihydroxyl and ortho-trihydroxyl structures, where two or more adjacent hydroxyl groups facilitate electron transfer and enhance redox activity. The presence of oxygen in the tumor environment can cause auto-oxidation of these phenols into semiquinones in a process where oxygen is reduced to O_2_^•−^ and leads to H_2_O_2_ production [41,55]. It has been observed that a pyrogallol-type structure in the B-ring (epigallocatechins, i.e., EGC and EGCG, R_2_ = OH) possesses H_2_O_2_-producing properties, which is responsible for its cytotoxic effect in Jurkat cells [45,56]. Hong et al. reported that a 50 μM dose of EGCG lead to generation of up to 25 μM of H_2_O_2_ in HT-29 human colon adenocarcinoma cells [57]. Among other antioxidants, EGCG produced the highest concentration of H_2_O_2_ at neutral pH in human oral tumor cell lines [46]. Nakagawa et al. suggested that a possible deprotonation or deprotonated form of EGCG in the pyrogallol moiety may contribute to H_2_O_2_ generation as the p*K*_a_ for EGCG is 7.59–7.75 [45].

**Figure 8:**
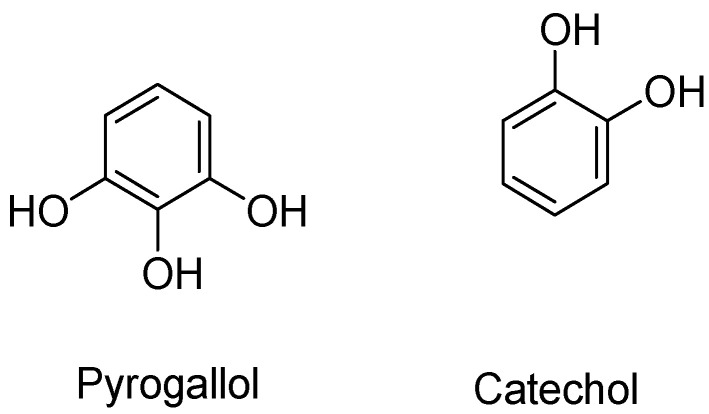
The structures of pyrogallol and catechol.

Pyrogallol itself has been shown to effectively generate H_2_O_2_ and O_2_^•−^ in various cell types, inducing O_2_^•−^ mediated cell death (Figure 8) [56]. Its concentration and incubation time affect intracellular H_2_O_2_ levels, with 100 μM pyrogallol causing a rapid and acute increase in H_2_O_2_ levels. The presence of pyrogallol reduces intracellular GSH content in HeLa cells, and the addition of Tempol, SOD, and CAT rescues cells from pyrogallol-induced apoptosis by increasing intracellular GSH content [58,59]. Miura also reported that flavonoids with a pyrogallol structure generated more H_2_O_2_ than flavonoids with a catechol structure [49]. For example, myricetin and baicalein demonstrated higher H_2_O_2_-generating abilities than quercetin and (−)-epicatechin. The distinct placement of hydroxyl groups introduces a capability to interact with several cellular components such as DNA, enzymes, proteins, and many others, which make these compounds more diverse in the field of medicine [60].

Findings reported by Nakagawa et al. suggested that the cytotoxic effects of flavonoids are not only due to a higher prooxidant ability to generate H_2_O_2,_ but also the cell’s ability to metabolize it [45]. Additionally, the production of flavonoid phenoxy radicals during antioxidative reactions can also generate prooxidant effects. These highly reactive phenoxy radical species undergo oxidation to generate flavonoid quinones that can conjugate with nucleophiles such as GSH, cysteine, or nucleic acids [61]. Phenoxy radicals of apigenin, naringenin, and naringin have been noted to rapidly oxidize NADH, leading to enhanced oxygen uptake and superoxide formation followed by H_2_O_2_ generation [62,63].

**Figure 9:**
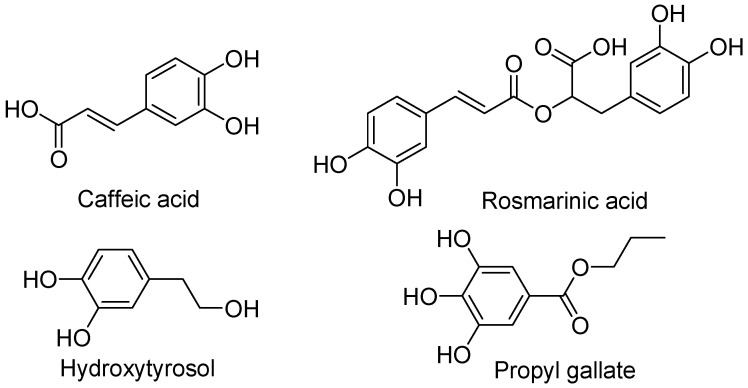
Polyphenols reported for their metal ion interactions to generate H_2_O_2_.

Besides autooxidation, many phenol analogues can interact with metal ions. Their metal chelation activity is mainly associated with the presence of ortho-dihydroxy groups [64]. These phenol compounds can be oxidized into semiquinones in the presence of metal ions via a one-electron transfer mechanism (Figure 6) [65], and along with reduced metal ions, react with O_2_, generating O_2_^•−^, oxidized metal ions and quinones. Involvement of these metal ions in redox reactions facilitate their regeneration and allow repeated cycles of redox reactions. Consequently, O_2_^•−^ accumulates and dismutates to produce H_2_O_2_ [40]. Figure 9 lists some examples of phenols that interact with metal ions to produce H_2_O_2_, such as caffeic acid, rosmarinic acid, hydroxytyrosol, and propyl gallate.

#### 4.1.2. Hydroxycinnamic Acid

**Figure 10:**
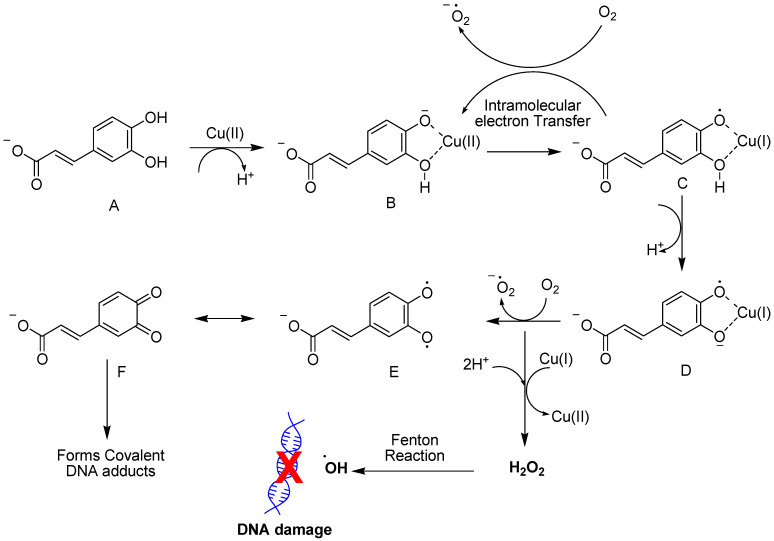
Mechanism of metal mediated H_2_O_2_ generation by caffeic acid (CA).

Caffeic acid (CA) and rosmarinic acid (RA) are hydroxycinnamic acid (HCA) analogues containing phenol moiety and are found in various dietary sources, including green tea, red wine, fruits, vegetables, and coffee, as well as in medicinal plants such as rosemary and salvia. Their extensive range of properties encompass anticancer, antioxidant, antiproliferative, and anti-inflammatory effects [66,67]. CA showed pro-oxidant potential due to its ability to interact with metals like copper, inducing lipid peroxidation and causing DNA damage within tumor cells through either oxidation or covalent adduct formation [68,69,70]. Zheng et al. proposed that the ortho-dihydroxyl groups can chelate Cu(II) to form a CA-Cu(II) complex A, therefore facilitating intramolecular electron transfer to generate a hydroxy phenoxy radical-Cu(I) complex C (Figure 10) [68,69]. During this process, CA undergoes deprotonation in response to copper, yielding a phenoxide anion that acts as a good ligand for metal ions due to the high electronic density of oxygen. Further deprotonation of C generated the semiquinone radical anion-Cu(I) complex D, which transfers an electron to O_2_ producing O_2_•^−^ and the final products, H_2_O_2_ and ortho-quinone. H_2_O_2_ can be subsequently converted into •OH via a Fenton reaction, inducing DNA damage. Ortho-quinone can form covalent adducts with the DNA of cancer cells. Similarly, RA with two diphenolic rings induces O_2_^•−^ and H_2_O_2_ formation in the presence of transition metals (i.e., iron) while producing the final product of o-quinones, which is corelated to the cytotoxicity of RA [71,72,73].

#### 4.1.3. Hydroxytyrosol

Hydroxytyrosol (HT), abundantly present in olives, has demonstrated anticancer properties in vitro [74,75]. Sun et al. demonstrated that HT exhibited antiproliferative and pro-apoptotic effects in cancer cells through H_2_O_2_ generation [75,76]. The mechanism of prooxidant activity of HT involves O_2_ and transition metals. First, the phenol moiety undergoes oxidation by Cu(II) or Fe(II), forming semiquinones, which then react with O_2_ generating O_2_^•−^ and finally producing H_2_O_2_ [77]. HT has been documented to generate H_2_O_2_ in colon cancer cells, ultimately leading to apoptotic cell death and mitochondrial dysfunction [75]. Similarly, in prostate cancer PC3 cells, HT has been linked to superoxide and H_2_O_2_ generation, triggering apoptosis [78,79]. Fabiani et al. have also reported that the chemo-preventive effects of HT rely on its prooxidant properties, hinging on its ability to generate H_2_O_2_ in the culture medium [80,81]. Their work has revealed that various amounts of H_2_O_2_ accumulate in the culture medium, influenced by its components and the cell’s ability to eliminate this peroxide. This clarifies the need for a wide range of HT concentrations to observe its chemo-preventive effects.

#### 4.1.4. Propyl Gallate

Propyl gallate (PG), chemically known as propyl-3,4,5-trihydroxybenzoate, is widely present in processed food and cosmetics, hair products, and lubricants [82,83,84,85]. This versatile compound boasts various biological properties, including potential antitumor effects. PG alone demonstrated antioxidative and cytoprotective properties against cellular damage and gained a pro-oxidative property in combination with copper (II) [86]. It was reported that PG was one of the most active compounds capable of generating H_2_O_2_ in DMEM media, which contributes to the cytotoxic effects observed in vitro [87].

Polyphenols are also widely used in many combination studies and possess a promising adjuvant property [38]. Several chemotherapeutic drugs have been shown to have significantly increased efficacy when combined with polyphenols. This includes cisplatin, 5-fluorouracil, celecoxib, doxorubicin, and tamoxifen. The synergistic effect between the two has been associated with drug resistance reduction, enhanced drug sensitivity, induction of cell cycle arrest, apoptosis, restricted angiogenesis, and anti-inflammatory and pro-oxidant effects [39,40].

### 4.2. Compounds with Quinone Moieties

**Figure 11:**
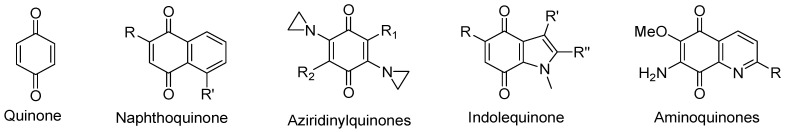
Structures of compounds containing quinone moiety that can induce H_2_O_2_ production via autooxidation and redox cycling.

**Figure 12:**
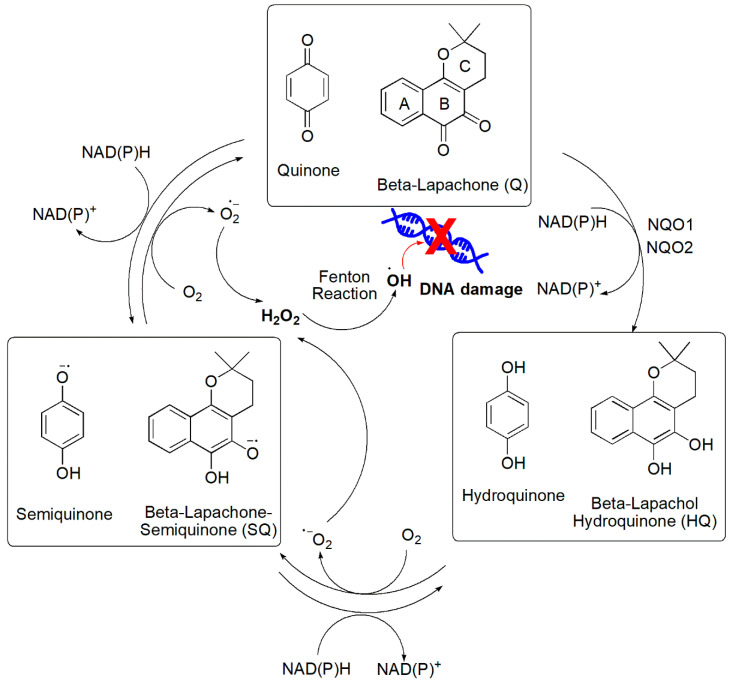
General mechanism of H_2_O_2_ generation by compounds (i.e., quinone and β-lapachone) containing quinone moiety via autooxidation and redox cycling pathways.

A wide range of quinone-containing compounds showed anticancer, antimicrobial and antiparasitic effects, such as naphthoquinones, aziridinylquinones, anthracyclines, indolequinones (i.e., mitomycins), aminoquinones (i.e., streptonigrins) and certain vitamins (Figure 11). H_2_O_2_ generation induced by these compounds is one of the possible mechanisms for their function. Quinones can induce H_2_O_2_ production in cells via autooxidation and redox cycling mechanisms. They undergo either one-electron reduction catalyzed by NAD(P)H-cytochrome P-450 reductase to form semiquinone radicals or two-electron reduction catalyzed by NAD(P)H:quinone oxidoreductase (NQO1 or NQO2) to generate hydroquinones (Figure 12). Semiquinone radicals and hydroquinones participate in redox cycling and undergo oxidation by O_2_ to regenerate quinones, while O_2_ is reduced to O_2_^•−^ that dismutates to form H_2_O_2_.

#### 4.2.1. Naphthoquinones

**Figure 13:**
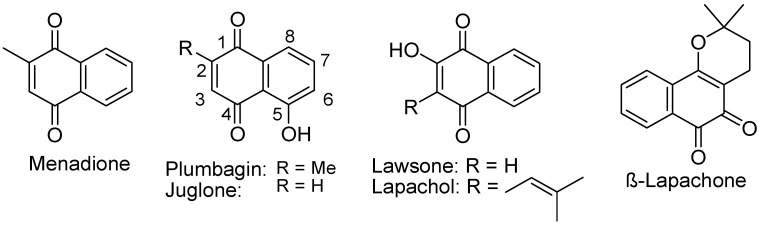
Structures of compounds containing naphthoquinone moiety that can induce H_2_O_2_ production through redox cycling.

Many naturally occurring naphthoquinones and their derivatives showed cytotoxicity, which has been investigated for the development of anticancer drugs, such as menadione (2-methyl-1,4-naphthoquinone, also termed vitamin K3), plumbagin, and juglone (Figure 13). Their toxic effects on cells are mostly caused by ROS species including H_2_O_2_ generated through redox cycling [88,89]. Criddle et al. has reported that menadione-induced ROS generation catalyzed by reductive enzymes, such as NADPH-cytochrome P-450 reductase, xanthine oxidase, and NQO1, promotes apoptosis of murine pancreatic acinar cells [88]. Menadione-induced ROS generation is concentration-dependent and high concentrations trigger cell death [90]. Clinical trials conducted on patients with prostate cancer showed that ascorbic acid-menadione produced an immediate drop in tumor cell numbers through a mechanism named autoschizis [91]. It has been proposed that autoschizis induced by ascorbic acid-menadione was caused by oxidative radicals generated by H_2_O_2_ leading to cellular damage.

#### 4.2.2. Hydroxyl Naphthoquinone

**Figure 14:**
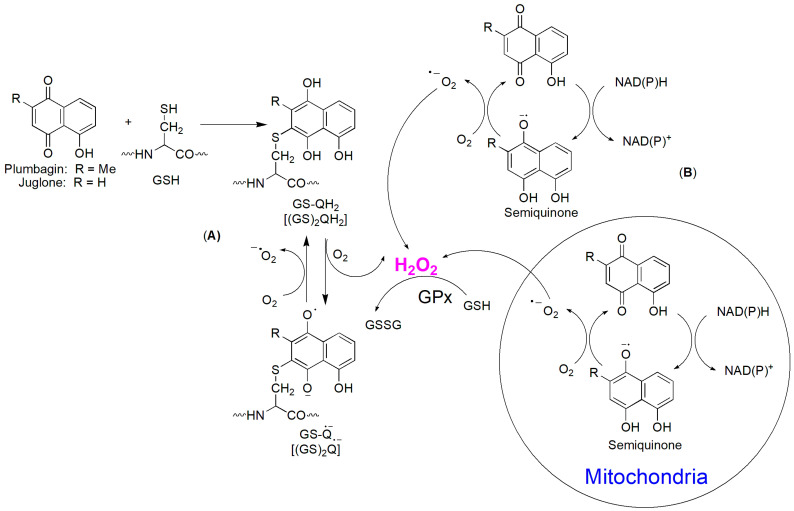
Two primary mechanisms for the cytotoxic effects of PL: (**A**) One-electron reduction of the quinone moiety generates semiquinone radicals that were engaged in redox cycling, generating both superoxide anions and H_2_O_2_ under aerobic conditions. (**B**) Direct interaction of the quinone moiety with thiol groups present in proteins and GSH causes the depletion of the reducing biomolecules and ROS amplification.

Plumbagin and juglone are hydroxyl naphthoquinone derivatives found in various plants, such as plants of the Plumbago genus and black walnuts. They possess a wide range of pharmacological properties such as antioxidant, anti-inflammatory, antifungal, antibacterial, antidiabetic, and anticancer effects. Their cytotoxicity is caused by two possible mechanisms: redox cycling and reaction with GSH, which both result in generation of the corresponding semiquinone radical and O_2_^•−^, leading to DNA damage and oxidative stress [92,93,94,95]. For example, PL interferes with mitochondrial electron transport due to its structural similarity to ubiquinone (Coenzyme Q, CoQ), which lowers oxygen consumption while generating oxygen radicals. Inbaraj et al. indicated that exposure of plumbagin and juglone to HaCaT keratinocytes caused a concentration dependent reduction of cell viability, which was attributed to two primary mechanisms (Figure 14) [96]. First, plumbagin and juglone undergo one-electron reduction by enzyme NAD(P)H-cytochrome P-450 reductase or two-electron reduction by mitochondrial NADH-ubiquinone oxidoreductase, resulting in the formation of semiquinone radicals and hydroquinones [97]. Under aerobic conditions, the semiquinone radicals or the hydroquinones formed participate in redox cycling and induce the reduction of O_2_ to generate O_2_^•−^ and H_2_O_2_. Second, quinone functional groups can directly react with thiol groups in proteins and GSH, resulting in GSH depletion and cell death [96]. It was found that a hydroxyl group at the C-5 position of naphthoquinones is correlated to heightened cytotoxicity due to improved redox cycling [98]. Plumbagin and juglone with a hydroxyl group at C-5 are much more reactive than lawsone and lapachol with an OH group in position C-2. Tautomerization of the C-2/C-3 enol structure of lawsone and lapachol will result in a saturated C-3 that prohibits Michael-type addition reactions in that position. This tautomerization also stabilizes the quinoid structure, which leads to a very low one-electron reduction potential, decreasing redox cycling [96].

#### 4.2.3. 1,2-Naphthoquinone

β-Lapachone (β-Lap), a 1,2-naphthoquinone natural product isolated from the lapacho tree, is a potent anticancer drug that has been advanced into clinical trials based on its tumor-selective cytotoxic properties [99]. Its antitumor mechanism is related to NQO1-mediated redox cycling. β-Lap and its derivatives undergo two-electron reduction catalyzed by NQO1 to form hydro-β-Lap (β-lapachol) which is highly reactive and unstable (Figure 12). It then undergoes auto-oxidation within the cell in two steps, first generating β-Lap-semiquinone that transforms into quinone-β-Lap (Q). This redox cycle produces O_2_^•−^ that dismutates into H_2_O_2_ [100]. Many studies indicated that β-Lap enhances H_2_O_2_ generation in cancer cells. Chau et al. reported that human leukemia cells treated with β-Lap had a substantial increase in intracellular H_2_O_2_, especially the ones with lower levels of GSH, including HL-60, U937, and Molt-4 [101]. The generated ROS have been linked to different pathways to apoptosis. It has been noted that the ROS generated by β-Lap resulted in the oxidation of ubiquitin specific protease 2 (USP2), which is known to protect tumor cells from apoptosis by preventing protein degradation. This oxidation happens by transforming its thiol groups to cysteine sulfinic or sulfonic acids. The deactivation of USP2 by β-Lap triggers proteasomal degradation pathways that contribute towards β-Lap-induced anticancer effects [99].

β-Lap consists of a benzene ring (A ring), an ortho-quinone ring (B ring), and a dihydropyran ring (C ring). Modifications have been made to A-, B-, and C-rings, resulting in a wide variety of promising derivatives with enhanced specificity and safety profiles [99]. Various derivatives of β-Lap have entered Phase I and Phase II clinical trials, either as a monotherapeutic agent or in combination with other cancer drugs. However, its rapid elimination and low bioavailability due to poor water solubility have posed challenges. Many derivatives such as ARQ 761, designed to overcome water solubility issues, also entered Phase I trials but exhibited only moderate potency [102]. MB12066, a derivative with an undisclosed structure, activated mitochondrial metabolism through NQO1 and entered Phase I and Phase II trials, demonstrating good safety profiles. However, these trials were ultimately terminated [103]. These trials aimed to explore the clinical potential of these β-Lap derivatives in cancer treatment, highlighting the importance of addressing solubility and potency challenges. These structural properties and modifications hold considerable promise for a range of biomedical applications, including cancer therapy, treatment of Chagas disease and tuberculosis, development of antifungal and antibacterial agents, and antimalarial drugs, ultimately contributing to advances in these critical fields of medicine [99].

#### 4.2.4. Anthracyclines

**Figure 15:**
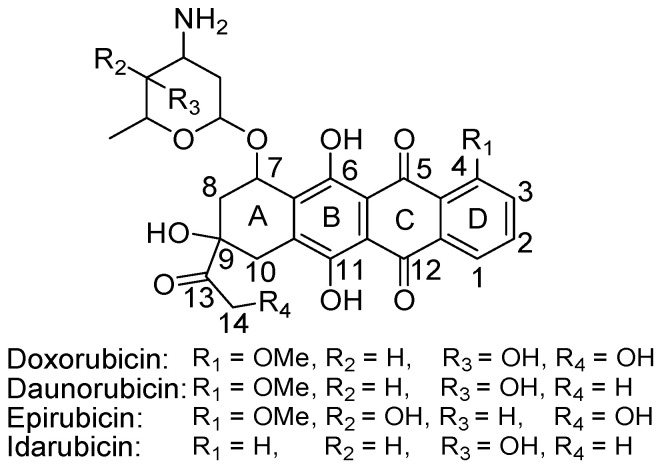
Structures of anthracyclines commonly used for cancer treatment.

**Figure 16:**
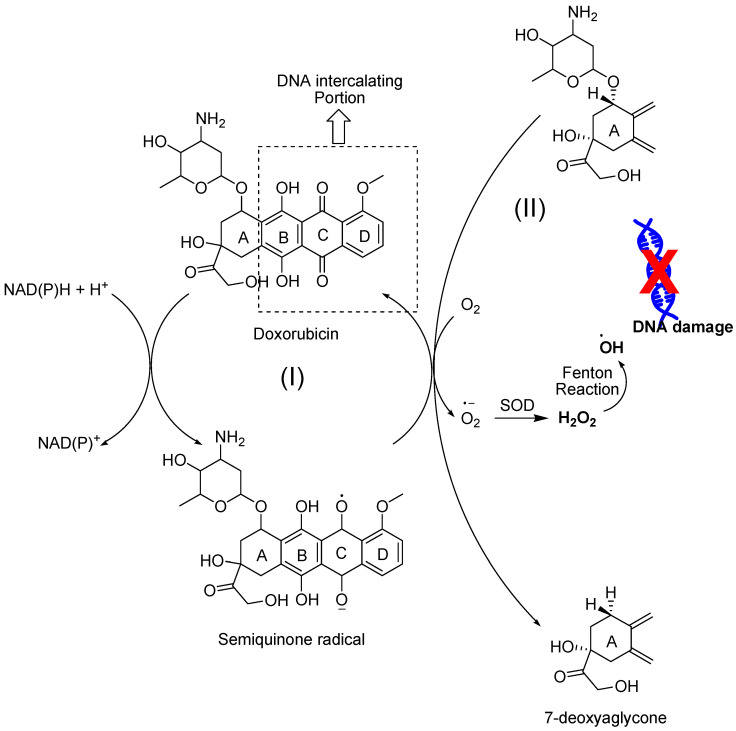
Two plausible mechanisms for ROS production induced by anthracyclines: (I) one-electron reduction of ring C generates a semiquinone radical, which triggers redox cycling catalyzed by NAD(P)H-oxidoreductases generating O_2_^•−^ and H_2_O_2_; (II) Reductive deglycosidation forms 7-deoxyaglycone with increased lipid solubility that intercalates into membranes producing site-specific ROS.

Some naphthoquinone-containing compounds, such as anthracyclines, are FDA-approved anticancer agents. Anthracyclines are among the most effective anticancer drugs ever developed. Doxorubicin (DOX) and daunorubicin (DNR) were the first anthracyclines that were isolated from Streptomyces peucetius bacteria (Figure 15) [104], and are commonly used for the treatment of both hematologic and solid tumors, such as breast cancer, childhood solid tumors, soft tissue sarcomas, aggressive lymphomas, and acute leukemias. Anthracyclines share a common structural framework characterized by a tetracyclic ring containing adjacent quinone-hydroquinone groups in rings B-C, a methoxy group at C-4 in ring D, and a short side chain at C-9 with a carbonyl at C-13. In addition, a sugar molecule called daunosamine is attached to the C-7 of ring A via a glycosidic bond [105,106,107,108]. Inducing oxidative damage in tumor cells has been considered an important mechanism for the anticancer effect of anthracyclines [105]. There are two mechanisms for superoxide production by anthracyclines (Figure 16). First, these drugs alter the properties of endogenous respiratory chain components, making them more susceptible to autooxidation by molecular oxygen [109]. The quinone moiety in ring C undergoes one-electron reduction to form a semiquinone that quickly regenerates its parent quinone by reducing O_2_ to O_2_^•−^ and H_2_O_2_. During this cycle, the glycosylic bond between ring A and daunosamine can also undergo reductive deglycosidation, leading to the formation of 7-deoxyaglycone (Figure 16). 7-deoxyaglycone has increased lipid solubility that allows for intercalation into biologic membranes and site-specific ROS production. One-electron redox cycling of DOX is also accompanied by a release of iron from intracellular stores which leads to the formation of drug–iron complexes that convert H_2_O_2_ into more potent hydroxyl radicals by a Fenton reaction. Second, due to their quinone nature, anthracyclines can function as artificial electron acceptors, withdrawing electrons from the respiratory chain. This action can lead to non-enzymatic oxidation of reduced anthracycline by O_2_, resulting in superoxide production. Superoxide produced in this process can contribute to the formation of H_2_O_2_ via dismutation [108].

Efforts to enhance anthracycline drugs have led to the development of around 2000 analogs, but with only a few advancing to clinical use [105]. Notable alternatives to DOX and DNR include epirubicin (EPI) and idarubicin (IDA) (Figure 15). EPI, derived from DOX, features an alteration in the hydroxyl group at C-4 in daunosamine, primarily affecting pharmacokinetics. Despite changes in volume of distribution and a shorter half-life, EPI can be used at higher cumulative doses without increased cardiotoxicity. IDA, derived from DNR, exhibits activity against various cancers, attributed to increased lipophilicity and improved stabilization of the drug–topoisomerase II-DNA complex [110].

#### 4.2.5. Aziridinylquinones

**Figure 17:**
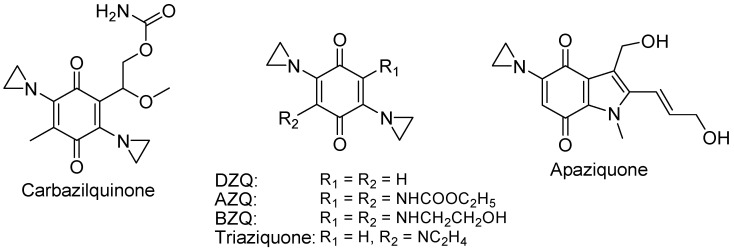
Structures of aziridinylquinones with an aziridine ring attached to a quinone group.

**Figure 18:**
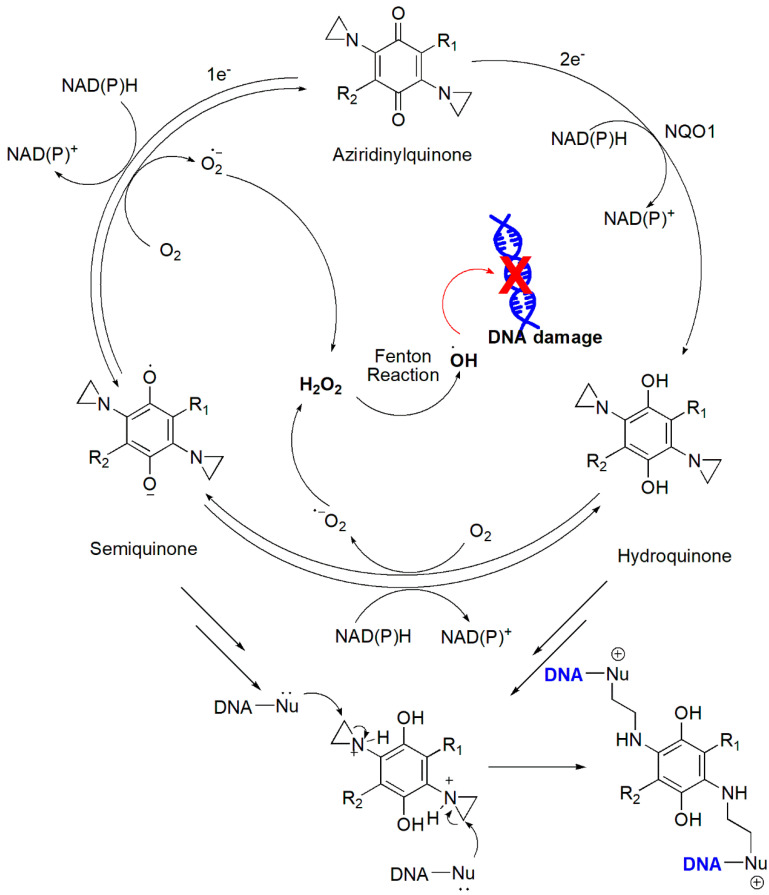
Possible mechanisms for the cytotoxic effects of aziridinylquinones: (A) Enzyme-catalyzed reduction leads to hydroquinone formation and redox cycling of semiquinone radicals, generating superoxide radicals and H_2_O_2_ under aerobic conditions. (B) Aziridinylquinones possess dual abilities, causing DNA damage and cell death via ROS and DNA-alkylation by aziridinium ion.

Aziridinylquinones, such as carbazilquinone, diaziquone (AZQ), BZQ, triaziquone, and apaziquone, have a unique structural composition with an aziridine ring attached to a quinone group (Figure 17). These compounds possess the ability to alkylate DNA and generate ROS, both of which contribute to their cytotoxic effects [111,112]. Aziridinylquinones undergo enzymatic reduction within cells, leading to the transformation of the quinone into a hydroquinone variant, which results in an elevation of the p*K*_a_ of the nitrogen atom within the aziridine ring (Figure 18). The increased pK_a_ makes the aziridine nitrogen atom in the hydroquinone variant more easily protonated, forming a highly reactive aziridinium cation that is a powerful DNA alkylating agent [111]. Meanwhile, molecular O_2_ reduces to H_2_O_2_ and other ROS in the redox cycling of semiquinone radicals formed via the reduction of aziridinylquinones catalyzed by enzymes like NADPH-cytochrome P-450 reductase. Under aerobic conditions, these radicals undergo redox cycling, generating O_2_^•−^ and H_2_O_2_ [112]. Under hypoxic tumor conditions where the availability of O_2_ is limited, on the other hand, aziridinylquinones such as AZQ undergo activation through a two-electron reduction mechanism facilitated by enzymes like DT-diaphorase (NQO1), forming semiquinone radical anions, which subsequently undergo redox cycling to produce cytotoxic H_2_O_2_. The ability of AZQ to exploit hypoxic environments enhances its cytotoxic effects in tumor cells [113].

#### 4.2.6. Indolequinones

**Figure 19:**
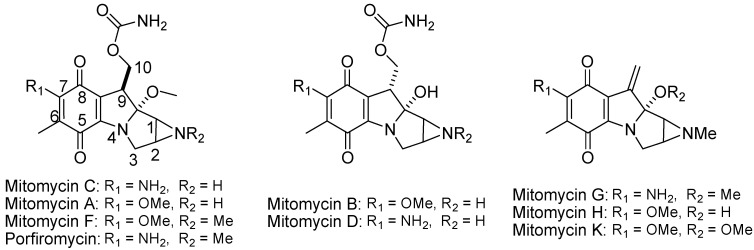
Structures of mitomycins that contain a complex tetracyclic pyrrolo-indole core adorned with an aziridine ring, carbamoyl group, and bridged carbinolamine, rendering them moderately stable under certain conditions but highly reactive with reducing agents.

**Figure 20:**
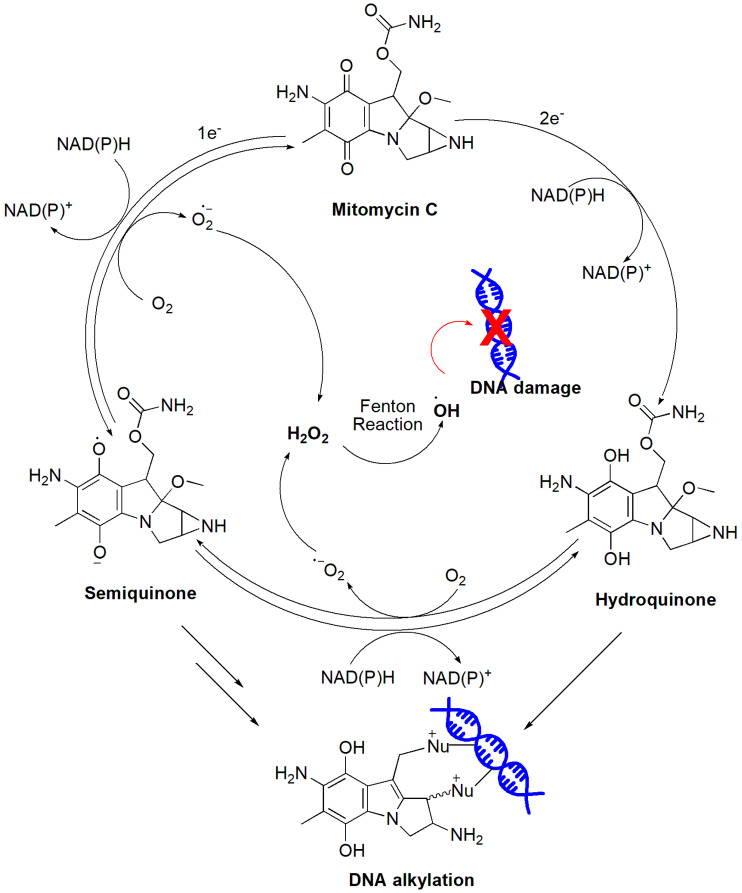
Proposed mechanism of mitomycins’ activity via H_2_O_2_ production and DNA adducts formation upon bioreduction.

Many naturally occurring indolequinone analogues, such as mitomycins (Figure 19), have shown potent anticancer properties [114]. They were recognized as prodrugs which undergo bioreduction in vivo to form irreversible bis-alkylation of DNA. The reduction of mitomycin initiates a reduction–oxidation cycle, which generates H_2_O_2_ as a byproduct [115]. Mitomycin C was the first member of this class of compounds to be discovered and was isolated from the fermentation broth of Streptomyces caespetosius. It demonstrates the most potent anticancer efficacy and has been used for the treatment of various tumors for decades [116]. Several mitomycin analogues have been identified with various modifications on the aziridine ring or quinone ring substituents, each retaining the biological activity of mitomycin C. Hydrophilic compounds, such as mitomycin C and porfiromycin, demonstrate the most effective anticancer effects against L1210 leukemia. The presence of an aziridine ring is essential for antileukemia activity, while quinone reduction potential strongly influences antibacterial activity (Figure 20) [117]. Among these mitomycin analogues, mitomycin C possesses unique chemical and physical properties, including good water solubility, low lipophilicity, and minimal binding to serum proteins, contributing to its potent anticancer properties. Correlations between partition coefficients and antitumor potency have been observed in some analogs, but correlations with quinone reduction potential or substituent size have been found to be insignificant in several studies [117,118].

#### 4.2.7. Aminoquinones

**Figure 21:**
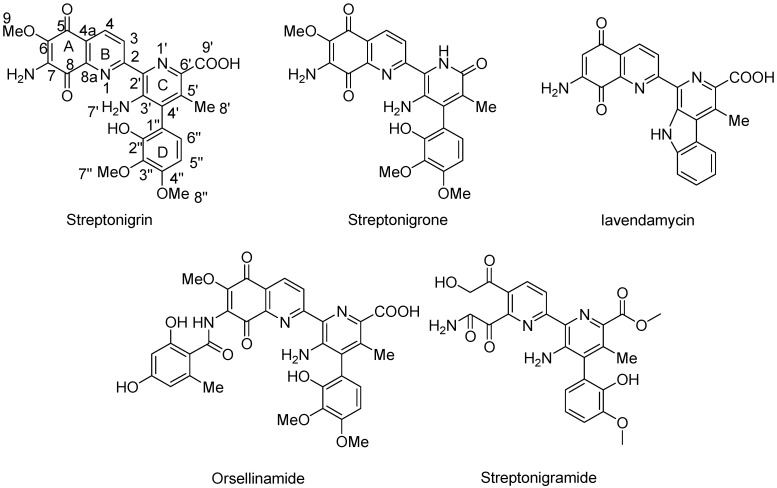
Structures of streptonigrin and its derivatives.

Streptonigrin and its derivatives contain aminoquinone moieties (Figure 21). They were isolated from Streptomyces flocculus and exhibit potent antitumor and antibiotic effects. Streptonigrin interacts with oxygen to generate superoxide radicals that undergo dismutation, producing H_2_O_2_ (Figure 12) [119]. The genotoxic effects of Streptonigrin are partly attributed to its ability to cleave DNA through a complex mechanism involving metal ions and autoxidation of its quinone moiety in the presence of NADH, leading to the production of oxygen-derived reactive species, including free radicals [119,120,121,122]. Recent studies have explored the involvement of free radicals in SN-induced DNA and chromosome damage. Antioxidant enzymes such as SOD and catalase, when added, prevent SN-induced DNA and chromosome damage [120,122]. Conversely, the hydroxyl radical scavenger mannitol intensified DNA and chromosome damage induced by SN [122]. However, when various antioxidants were encapsulated into liposomes and added to cell cultures, either alone or in combinations, a significant reduction in SN-induced chromosome aberrations and DNA damage was observed. This suggests that free radicals play a role in SN-induced genotoxicity and that this damage can be partially mitigated by incorporating antioxidants into cells [122]. Streptonigrin was previously used as an anticancer drug but has been discontinued because of its toxic effects. Analogues of SN, such as streptonigrone, lavendamycin, orsellinamide, and streptonigramide have been designed and investigated, which did not lead to better anticancer activity or selectivity (Figure 21).

### 4.3. Vitamin C

**Figure 22:**
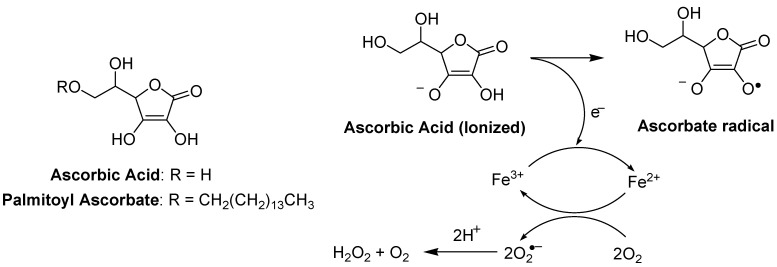
The structure of ascorbic acid and derivatives and the proposed mechanism for the formation of H_2_O_2_ induced by ascorbic acid.

Vitamin C (also known as ascorbic acid or ascorbate) is an essential vitamin in the body’s daily function. It allows for the biosynthesis of collagen and various neurotransmitters, is involved in protein metabolism, and strengthens the body’s immune system. In recent years, ascorbic acid has been shown to have selective anticancer properties at millimolar (mM) concentrations, with such an anticancer effect demonstrated both in vitro and in vivo [123,124,125,126]. The main mechanism through which vitamin C kills tumor cells is by formation of H_2_O_2_ [124,127]. At the beginning of this process, the ionized vitamin C is transformed into ascorbate radical by losing one electron (Figure 22). This electron then reduces a protein-centered metal, such as Fe^3+^ to Fe^2+^. The created Fe^2+^ then donates an electron to O_2_, forming O_2_^●−^ that is subsequently dismutated to form H_2_O_2_ and O_2_. The created H_2_O_2_ can cause damage to DNA, lipids, and proteins, inducing cancer cell death. Notably, these concentrations of Vitamin C are not enough to kill healthy, non-cancerous cells due to the high level of plasma catalase and/or GSH peroxidase that inhibit the redox reaction or destroy any formed H_2_O_2_ molecules, thus making cancer treatment via vitamin C even more appealing due to its selective nature.

Although early randomized clinical trials concluded that oral administration of high-dose vitamin C to patients with advanced cancers does not have any therapeutic benefits, parenteral (i.v. or i.p.) injection of vitamin C can result in concentrations as high as 70× (up to 20 mM) that of an oral dose in the plasma, a concentration high enough to damage cancer cells [128,129,130]. Currently, there are more than a dozen completed and active clinical trials investigating the effectiveness of high-dose vitamin C to treat cancer (https://clinicaltrials.gov (accessed on 1 June 2024)). Derivatives of vitamin C and combination therapy have also been developed to improve its efficacy. For example, hydrophobized palmitoyl ascorbate was used to construct polymeric micellar nanoparticles to further enhance anticancer efficacy and selectivity [131,132]. Another possible avenue that could be taken with vitamin C cancer treatment is using it in conjunction with other therapeutics, such as vitamin K3, triethylenetetramine, or other H_2_O_2_-responsive chemotherapeutic drugs (i.e., camptothecin) to achieve synergistic anticancer effect while minimizing unwanted side effects [133,134,135].

### 4.4. Metal, Metal Oxides, and Metal Peroxides

Metals play an essential role in biological systems and human health. Many enzymatic reactions require metals for their catalytic action [136]. Essential metals such as calcium, sodium, potassium, magnesium, and transition metals iron, copper, and zinc are vital as well. Deficiency or excess of these metals can cause various diseases including cancer [137]. Exposure to heavy metals like arsenic, cadmium, chromium, nickel, lead, and mercury, even at low levels, can be toxic and contribute to various cancers including skin and lung cancers. Although the molecular mechanism is not completely understood, their potential to generate ROS and alter cellular redox status is considered significant in metal-induced carcinogenesis [138]. On the other hand, many metals, metal complexes, or metal peroxides have gained significant attention in cancer treatment, which has been highlighted in several reviews [139,140,141]. Some metal oxides and peroxides are reported to enhance H_2_O_2_ production, which is one of the possible mechanisms for their anticancer efficacy and selectivity [141]. This section does not intend to give a comprehensive review on metals, their oxides, and peroxides, but instead aims to discuss examples of metal oxides and peroxides that directly generate H_2_O_2_ in cancer cells and to highlight their role in cancer treatment.

**Scheme 1:**
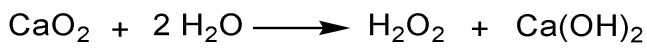
A reaction between metal peroxide and H_2_O to produce H_2_O_2_ using CaO_2_ as a representative example.

There are several pathways for metal oxides or peroxides to induce H_2_O_2_ production, including inhibition ofantioxidant enzymes, photocatalysis, or via a chemical reaction with water. For example, trisenox, also known as As_2_O_3_, induces H_2_O_2_ production by inhibiting GPx and catalase [142,143,144]. Titanium dioxide (TiO_2_) generates H_2_O_2_ primarily via photocatalysis [145,146]. Many metal peroxides, such as MgO_2_, CaO_2_ can react with H_2_O to produce H_2_O_2_. Such a reaction is facilitated under acidic conditions (Scheme 1) [140,141].

Many metals and their oxide forms have limited therapeutic potential due to metal carcinogenesis. Modification of metal oxides with less toxicity, e.g., titanium dioxide (TiO_2_) [145], zinc oxide (ZnO) [147], etc., into nanoparticle (NP) forms allows for the targeting of cancer tissues more accurately due to their smaller size and greater bioavailability. For example, TiO_2_ NPs can be activated upon UV irradiation to produce various ROS (i.e., H_2_O_2_), leading to cytotoxicity. This process has been applied in photodynamic therapy to treat cancer [145]. The popularity of ZnO has also risen due to being safe and efficient delivery [147,148], and being categorized as “generally recognized as safe” (GRAS) by the U.S. FDA (21CFR182.8991). Its functionality as an antibacterial and anticancer agent primarily relies on its ability to generate ROS [147,149]. A wide variety of ZnO NPs have been developed, which showed selective cytotoxicity towards cancer cells. The ZnO NPs undergo low-pH-dependent dissolution into Zn^2+^ ions, which can disrupt cell membrane and mitochondrial functions, inducing ROS generation and leading to cancer cell death.

Metal peroxides (MO_2_) have also gained popularity recently due to their ability to react with water to form H_2_O_2_ which is facilitated under acidic conditions (Scheme 1). Many metal peroxide NPs have been constructed to target unique tumor microenvironments, including hypoxia, low acidity, and high H_2_O_2_ and GSH levels. Such NPs include ones containing CaO_2,_ MgO_2_, BaO_2_, ZnO_2_, or CuO_2_ [141,147,149,150]. For example, Zhang’s group constructed a CaO_2_-based nanocatalytic medicine, which simultaneously supplies O_2_ and H_2_O_2_ to achieve enhanced chemo/chemodynamic therapy [151]. Tang and co-authors developed a biodegradable transferrin-modified MgO_2_ nanosheet that produced large quantities of H_2_O_2_ selectively in cancer cells in response to the acidic and low catalase activity of the tumor microenvironment [141]. Chen’s group reported a method of fabricating CuO_2_ nanodots which are sensitive to the acidic environment of tumor cells, leading to simultaneous release of H_2_O_2_ and Cu^2+^ [152]. These tumor-targeting metal-peroxide NPs showed enhanced tumor growth inhibition and minimal side effects in vivo.

Among various metal peroxides, CaO_2_ shows the most promise due to its biocompatibility and potential for use in cancer treatments like calcium overload therapy and treatment of bone-related cancers. In catalytic medicine, H_2_O_2_ can be utilized to generate large amounts of hydroxyl radicals through a Fenton-like reaction. MO_2_ has been found to be effective in enhancing therapeutic effectiveness in procedures that involve O_2_, such as photodynamic therapy and radiotherapy. Thus, metal peroxide-based nanoparticles are emerging as a novel avenue for cancer treatment. They have been extensively investigated in the field of biomedical science for their H_2_O_2_ and O_2_ generation capabilities.

### 4.5. FDA-Approved Drugs

**Figure 23:**
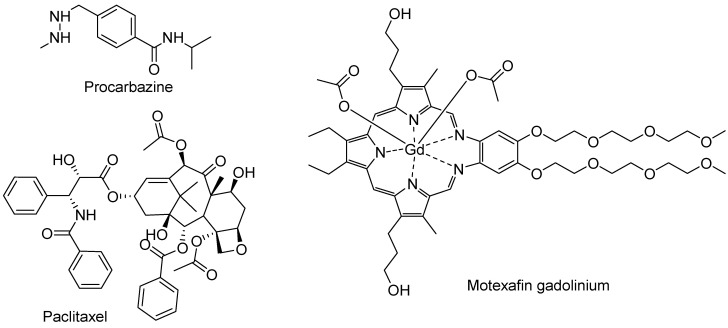
FDA-approved drugs that generate H_2_O_2_.

Many FDA-approved anticancer drugs not belonging to categories already touched on also undergo oxidation and induce O_2_ reduction to generate O_2_^●−^ and H_2_O_2_. These drugs include procarbazine, paclitaxel, motexafin gadolinium, and others (Figure 23).

**Figure 24:**
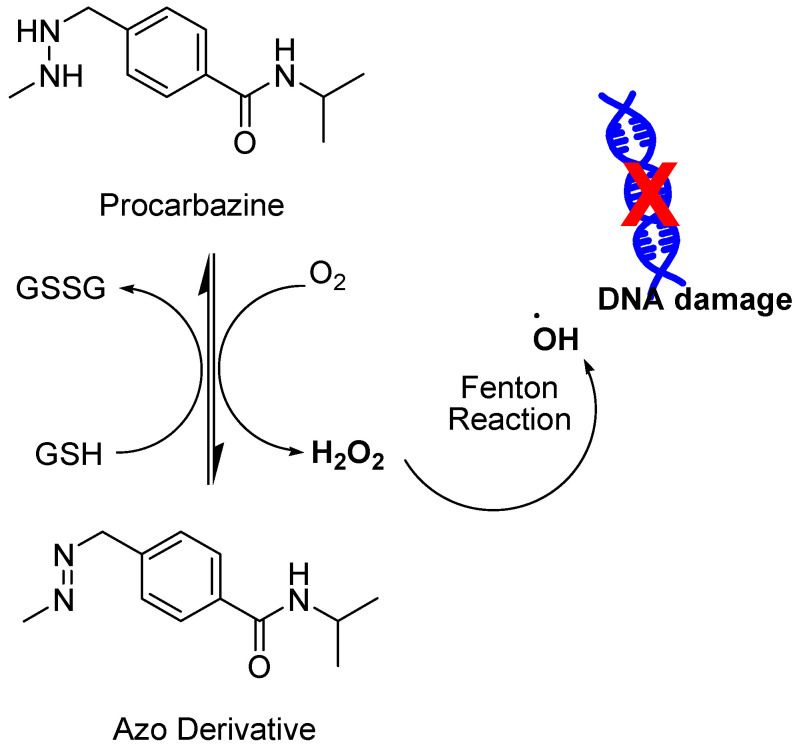
Oxidation of procarbazine to azo derivative generates H_2_O_2_ that subsequently undergoes Fenton reaction to form OH^•^ causing DNA damage.

Procarbazine is a hydrazine derivative that is widely used for the treatment of various types of cancer, including Hodgkin’s lymphoma, non-Hodgkin’s lymphoma, and primary brain tumors [153]. It was one of the first drugs to be developed that generates ROS, notably H_2_O_2_, in order to combat cancer cells [154]. When exposed to oxygen, procarbazine undergoes oxidation and triggers the reduction of O_2_ to form H_2_O_2_, which is combined with iron (Fe^2+^) to produce OH•, causing damage to cellular components, such as DNA (Figure 24) [154,155]. Procarbazine can cross the blood–brain barrier, which has made it a valuable treatment for primary brain tumors [154,155]. In clinical applications, procarbazine is mostly administered in combination with other drugs to treat Hodgkin’s lymphoma, non-Hodgkin’s lymphoma, and specific primary brain tumors. It has also shown remarkable efficacy in regimens like MOPP (mechlorethamine, vincristine, procarbazine, and prednisone) when used to treat Hodgkin’s lymphoma [156,157].

Paclitaxel (PTX), also known as taxol, was the first microtube stabilizing agent widely used in chemotherapy. Its primary mechanism of action is to bind and stabilize microtubules and inhibit cell division [158]. Recently, it has been found that paclitaxel cytotoxicity is also correlated with ROS production [159]. PTX has been shown to induce excessive production of O_2_^•−^ and H_2_O_2_, leading to oxidative stress in various cancer cell types, such as lung and breast cancer cells. This process may involve activation by NADPH oxidases that play a role in generating O_2_^•−^ from oxygen and NADPH [160]. Paclitaxel-induced stabilization of microtubules within cells could potentially trigger the activation of NOX via a pathway that involves Rac GTPase, which is known to closely interact with microtubules. Therefore, paclitaxel’s impact on microtubules might influence Rac GTPase activity, subsequently activating NOX and leading to O_2_^•−^ production [161,162]. Spitz et al. have demonstrated that combination of PTX with inhibitors of glucose and H_2_O_2_ metabolism greatly elevate H_2_O_2_ levels, which enhances killing of breast cancer cells [163]. The clinical implications of these findings are profound. Combining inhibitors of glucose and H_2_O_2_ metabolism with PTX could represent a novel strategy to amplify oxidative stress selectively in cancer cells, making them more susceptible to cytotoxicity while minimizing harm to normal cells. This approach holds potential for improving the therapeutic efficacy of PTX, especially in breast cancer treatment [163].

**Figure 25:**
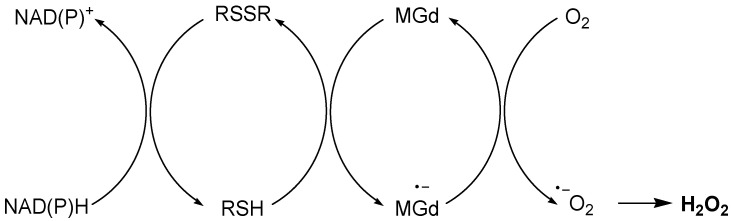
MGd induces H_2_O_2_ production via transferring electrons from biological reducing agents, such as protein thiols, thioredoxin, and glutathione, to form MGd radical anion that reduces O_2_ to O_2_^•−^ and form the final product H_2_O_2_.

Motexafin gadolinium (MGd) is a gadolinium texaphyrin complex that has a strong affinity for electrons. MGd can accept electrons from various reducing metabolites, such as protein thiols, thioredoxin, nicotinamide adenine dinucleotide phosphate (NADPH), and GSH, and transfer them to oxygen, resulting in the production of O_2_^•−^ (Figure 25) [164]. This electron transfer process interferes with ATP production and promotes apoptosis [165]. MGd is specifically designed to localize within tumor cells, targeting cancerous tissue due to its affinity for the abnormal metabolic processes found in these cells. In contrast to normal cells, cancer cells predominantly employ anaerobic glycolysis for their energy production. This abnormal metabolism provides a crucial distinction for the drug’s action. Once inside cancer cells, MGd initiates a unique mechanism where electrons are transferred from reducing metabolites within the cancer cells to O_2_, generating superoxide anions that are disproportionate to form oxygen and H_2_O_2_ [164]. Crucially, H_2_O_2_ and other ROS produced during this process are selectively trapped within tumor cells, resulting in damage to cellular DNA, proteins, and lipids inside cancer cells, eventually leading to apoptosis [164].

### 4.6. H_2_O_2_ Concentrations Induced by Representative Compounds

H_2_O_2_ concentrations produced by various compounds can provide valuable insights into their potential efficacy and mechanisms of action. Several methods have been used for measurement of H_2_O_2_ concentrations, including Amplex Red Assay Kit, ferrous ion oxidation—xylenol orange method, 2′,7′-dichlorofluorescein diacetate (DCFH2-DA), boronate fluorophore peroxyxanthone (PX1), and oxygen electrode, etc. Here, we summarize the reported H_2_O_2_ concentrations induced by some representative compounds discussed in this review and the corresponding methods employed, which aims to offer a clear picture for the possible level of H_2_O_2_ that can be achieved in tumor tissue and inside cells (Table 1). However, H_2_O_2_ generation depends on numerous factors such as the specific compounds used, cancer cell type, microenvironment, assays used for measurement, experimental conditions etc.

## 5. Conclusions

Redox adaptations among cancer cells have been characterized by their heightened oxidative stress thresholds as well as their increased intracellular antioxidant defenses. To push cancer cells beyond their cytotoxic threshold necessitates approaches that disrupt these adaptations. One such approach is the manipulation of ROS, particularly H_2_O_2_. This can be achieved in a few ways, including inhibition of endogenous antioxidants, modulation of proteins responsible for maintaining redox homeostasis, and use of compounds that generate H_2_O_2_ selectively within cancer cells. The strategy to selectively enhance H_2_O_2_ levels in cancer cells has emerged as a potential therapeutic approach. Vulnerabilities within cancer cells make them susceptible towards H_2_O_2_-mediated toxicity or growth inhibition mechanisms. This review has illuminated the diverse roles of phenol and polyphenol analogues, along with quinone-containing compounds, in generating H_2_O_2_ via autooxidation and redox cycling processes. These mechanisms entail continuous interconversion between phenolic compounds and their quinone forms, resulting in the production of O_2_^•−^ that dismutates into H_2_O_2_ to induce malignant cell death. Compounds like epigallocatechin gallate (EGCG) and hydroxytyrosol (HT) have demonstrated substantial anticancer effects through their prooxidant activities. β-Lapachone’s NQO1-mediated redox cycling yields significant amounts of H_2_O_2_, leading to oxidative damage and apoptosis. Moreover, the interaction of these compounds with metal ions further enhances their prooxidant activities, as observed with hydroxycinnamic acids such as caffeic acid and rosmarinic acid. The metal-chelating properties of these compounds facilitate additional redox reactions, amplifying H_2_O_2_ production and augmenting their anticancer effects. Aziridinylquinones and indolequinones, with their unique structural properties, have shown promise in generating ROS and inducing DNA damage, enhancing their cytotoxic effects on cancer cells. Vitamin C, particularly when administered parenterally at high doses, has demonstrated targeted anticancer properties by inducing oxidative stress selectively in tumor cells. Current clinical trials and combination therapies aim to capitalize these properties to enhance therapeutic outcomes. The utilization of metal oxides and metal peroxides has introduced novel mechanisms for inducing oxidative stress in cancer cells. Nanoparticles of metal oxides or peroxides, particularly ZnO and TiO_2_, have shown enhanced targeting effect and reduced side effects, holding promise for future cancer therapies.

Various FDA-approved drugs like procarbazine, paclitaxel, and motexafin gadolinium highlight the critical role of ROS in cancer treatment. However, there are still challenges that must be overcome before H_2_O_2_-amplifying agents can be used for targeted cancer therapies, such as ROS heterogeneity, targeted drug delivery, the difficulty of achieving optimal selectivity, safety, and toxicity concerns. Researchers have been addressing these challenges by combining H_2_O_2_-generating agents with other chemotherapeutic agents, nanoparticles, or other prooxidant-based therapies, for an effective and personalized cancer treatment. We recently observed that combination of H_2_O_2_-generating prooxidants with H_2_O_2_-activated prodrugs has potential to enhance anticancer efficacy and lower the required dosage, which in turn reduces the risk of toxicity towards healthy tissues [174]. Understanding the functions and mechanisms of these H_2_O_2_-generating compounds facilitates the design and development of innovative approaches to combat cancer, which offer improved therapeutic outcomes. The insights gleaned from these studies underscore the dual nature of these compounds as both antioxidants and prooxidants, offering a strategic advantage in cancer therapy. Future research endeavors should focus on optimizing the balance between prooxidant and antioxidant activities to maximize therapeutic benefits while minimizing adverse effects. The structural versatility and redox properties of these compounds hold immense promise for developing effective anticancer strategies and enhancing the efficacy of existing chemotherapeutic agents.

## Figures and Tables

**Table 1:** Reported H_2_O_2_ concentrations for representative compounds.

Prooxidant	Dose	Cell Line	H_2_O_2_ Produced	Method	Ref.
Phenols and polyphenol analogues					
EGCG	50 µM	Jurkat	~1 µM	Ferrous ion oxidation--xylenol orange method	[45]
	10 µM	HCT116 and HT29	1.5 µM	Amplex Red Assay Kit	[166]
	100 µM	Jurkat	20 µM	Hydrogen Peroxide Quantitative Assay Kit from Sangon Biotech	[167]
Quercetin	70 µM	HT29	2 µM	Amplex Red assay kit	[168]
Compounds with quinone moieties					
Menadione	10 µM	Jurkat	20 µM	2′,7′-dichlorofluorescein diacetate (DCFH2- DA)	[169]
Plumbagin	4 µM	SiHA and HeLa	1 mM	DCFH2-DA	[170]
β-Lap	1 µM	HL-60	70 µM	DCFH2- DA	[101]
Anthracyclines					
Doxorubicin	1 µM	PC3	38 pM	Amplex Red Assay Kit	[108]
Vitamin C					
Ascorbic Acid	1 mM	HL-60	161 µM	Oxygen electrode	[171]
	0.2–2.0 mM	Burkitt’s lymphoma	20–120 µM	Oxygen electrode	[123]
	i.v. 0.5 mg/g	rats	0–20 µM	Boronate fluorophore peroxyxanthone (PX1)	[124]
	i.p. 4.0g/kg	Mice tumor	>125 µM	PX1	[125]
Metal, metal oxides, and metal peroxides					
TiO_2_	10 µg/mL	HepG2	150 nmol/mL	DCFH-DA assay	[172]
FDA-approved drugs					
Paclitaxel	100 nM	MCF7	600 nM	Amplex Red Assay Kit	[173]
	100 nM	HL-60	1100 nM	Amplex Red Assay Kit	[173]

## Data Availability

No new data were created or analyzed in this study.
